# Sanitary Conditions Affect the Colonic Microbiome and the Colonic and Systemic Metabolome of Female Pigs

**DOI:** 10.3389/fvets.2020.585730

**Published:** 2020-10-26

**Authors:** Marinus F. W. te Pas, Alfons J. M. Jansman, Leo Kruijt, Yvonne van der Meer, Jacques J. M. Vervoort, Dirkjan Schokker

**Affiliations:** ^1^Wageningen Livestock Research, Wageningen University and Research, Wageningen, Netherlands; ^2^Department of Agrotechnology and Food Sciences, Biochemistry, Wageningen University, Wageningen, Netherlands

**Keywords:** pig (Sus scrofa), high and low sanitary conditions, colonic microbiota, colonic and systemic metabolome, pig, health status, colonic microbiome, metabolome

## Abstract

Differences in sanitary conditions, as model to induce differences in subclinical immune stimulation, affect the growth performance and nutrient metabolism in pigs. The objective of the present study was to evaluate the colonic microbiota and the colonic and systemic metabolome of female pigs differing in health status induced by sanitary conditions. We analyzed blood and colon digesta metabolite profiles using Nuclear Magnetic Resonance (1H NMR) and Triple quadrupole mass spectrometry, as well as colonic microbiota profiles. 1H NMR is a quantitative metabolomics technique applicable to biological samples. Weaned piglets of 4 weeks of age were kept under high or low sanitary conditions for the first 9 weeks of life. The microbiota diversity in colon digesta was higher in pigs subjected to low sanitary conditions (*n* = 18 per treatment group). The abundance of 34 bacterial genera was higher in colon digesta of low sanitary condition pigs, while colon digesta of high sanitary status pigs showed a higher abundance for four bacterial groups including the *Megasphaera* genus (*p* < 0.003) involved in lactate fermentation. Metabolite profiles (*n* = 18 per treatment group) in blood were different between both groups of pigs. These different profiles suggested changes in general nutrient metabolism, and more specifically in amino acid metabolism. Moreover, differences in compounds related to the immune system and responses to stress were observed. Microbiome-specific metabolites in blood were also affected by sanitary status of the pigs. We conclude that the microbiome composition in colon and the systemic metabolite profiles are affected by sanitary conditions and related to suboptimal health. These data are useful for exploring further relationships between health, metabolic status and performance and for the identification of biomarkers related to health (indices) and performance.

## Introduction

Management conditions, such as sanitary conditions, can affect pig health, and productivity (1, 2). Therefore, induced variation in sanitary conditions can be used to study the effects of subclinical health status on nutrient metabolism and requirements. Low sanitary conditions induce activation of the immune system which can influence amino acid (AA) metabolism and energy requirements (3, 4). The immune system metabolism synthesizes specific proteins. This means both specific AA requirements for these proteins and energy consumption for the metabolism. Kahindi et al. (5) showed that the sulfur amino acid to lysine ratio differed between sanitary conditions. Furthermore, the sanitary status related health status may negatively affect the growth response of the animal to nutrient intake. This may be due to gut morphology changes and body metabolic changes measurable as plasma urea nitrogen content (6). Sanitary conditions not only affect performance, but also modify the behavior of animals (7). The optimal dietary AA concentrations and profile depend on animal (e.g., genetics, immune or metabolic status) and environmental conditions (e.g., environmental temperature or pathogen pressure) (5, 8). In addition, repartitioning of dietary protein and AAs from development and growth of tissues (body protein deposition) to processes related to immune system activation can also affect the pig's AA metabolism (5, 9). Immune stimulation is often followed by a reduction in feed intake. As a consequence pig performance can be reduced as a result of immune system activation by low sanitary conditions or specific immune challenges (7).

Gut health is important for the digestive function of the gut consisting of enzymatic and fermentative hydrolysis of nutrients and their subsequent absorption from the lumen into the gut tissue and systemic circulation [reviewed in Pluske et al. (10)]. The gut microbiome composition is a major regulatory factor underlying nutrient digestion and fermentation, in particular in the hindgut (10). Management, e.g., sanitary conditions, may affect gut health via both the gut microbiome composition and its metabolic activity, which may in turn also affect the availability of nutrients resulting from fermentation, which contributes to the animal's productivity (11, 12). Thus, sanitary conditions may affect health status of the animal, which influences the microbiome composition and interfere with enzymatic and fermentative nutrient digestion, thereby potentially influencing production traits. Van der Meer et al. (12) studied the performance and immune status of pigs kept under different sanitary conditions. They showed that the growth performance of the pigs was influenced by dietary protein supply (adequate vs. restricted). Furthermore, a link between damaging behavior, sanitary conditions, and dietary protein supply was reported, indicating that animals showed more damaging behavior under conditions of protein restriction and low sanitary status (13).

The nutrient composition of intestinal digesta is dependent of diet composition and processes of nutrient hydrolysis, fermentation, and absorption in the gut (14, 15). Related complex changes in composition can be studied by measuring changes and differences in the gut metabolome (16, 17). Metabolomics offer the possibility to study many metabolites simultaneously in a single sample (18). It is not yet possible to study the entire metabolome in a sample using a single methodology. Therefore, we used Nuclear Magnetic Resonance (NMR) and Triple quadrupole mass spectrometry (TQMS) to study the metabolite profiles in colon digesta and blood, respectively (19, 20). In addition, we studied the microbiome composition in the colon digesta (21). Together, this approach provides data on the gut metabolic activity and systemic metabolic status of pigs. In a previous report we showed the relationship between animal health status and productivity of the pigs used in the present study (4).

The objective of this study was to explore the colonic microbiota and metabolome and the systemic metabolome of pigs differing in health status induced by imposed sanitary conditions. Such approach can be useful for further exploring relationships between health, metabolic status and performance in pigs, including identification of predictive biomarkers for health and performance.

## Materials and Methods

### Animal Experimental Design

The experimental design of the animal study and the sanitary challenge model used have been described by Van der Meer et al. (4). Shortly, 144 female piglets were selected at 1week of age on a commercial farm in The Netherlands and used in 3 subsequent batches of 48 pigs. Within each batch half of the piglets were selected for LSC and the other half for HSC treatment. Table 1 describes the composition of the diet fed *ad libitum* to the animals. As part of the challenge model, vaccination against a number of relevant pathogens was only given to high sanitary condition (HSC) pigs and not to low sanitary condition (LSC) pigs. At the first week of age high sanitary condition (HSC) pigs were vaccinated against Mycoplasma hyopneumoniae (Porcilis M Hyo, MSD Animal Health, Boxmeer, The Netherlands) by subcutaneous injection in the neck. Upon arrival and 2 days thereafter, HSC pigs received an antibiotic injection (Fenflor, AUV, Cuijk, The Netherlands, one mL per pig, intramuscular per time point). In week four of age HSC pigs were vaccinated against Mycoplasma hyopneumoniae, Porcine circovirus type 2, Porcine Reproductive and Respiratory Syndrome (PRRS), and Lawsonia intracellularis (Porcilis M Hyo, Porcilis Circo, Porcilis PRRS, all MSD Animal Health, Boxmeer, The Netherlands, and Enterisol Ileitis, Boehringer Ingelheim, Alkmaar, The Netherlands). At 6 weeks of age HSC pigs were vaccinated against Actinobacillus pleuropneumoniae, and Influenza A virus (Porcilis APP, MSD Animal Health, Boxmeer, The Netherlands, and Gripovac3, Merial, Velserbroek, The Netherlands); and at 8 weeks of age HSC pigs were vaccinated against Actinobacillus pleuropneumoniae, and Influenza A virus (Porcilis APP, MSD Animal Health, Boxmeer, The Netherlands, and Gripovac3, Merial, Velserbroek, The Netherlands) by subcutaneous injection in the neck or in case of Enterisol by oral drench. After weaning at 3 weeks of age the piglets were transferred to the experimental location. The experimental treatments started at arrival and lasted for a period of 10 weeks. During the final 4 weeks of the period, the animals were kept in climate respiration chambers for measurements on energy metabolism of the pigs. The temperature in the rooms was set at 24°C at the start of the experiment and was decreased to 20°C during the experiment. In contrast to rooms for HSC pigs, the low sanitary condition (LSC) rooms were not cleaned after the previous batch of commercial finisher pigs that left the facility 2 days before, and no specific hygiene protocol was applied upon entering the rooms or during execution of procedures applied to the pigs in these rooms. An equal mixture of fresh manure from three commercial pig farms was spread in the LSC pens every week during the final 4 weeks of the experimental period to enhance antigenic pressure. In contrast, HSC pigs received a dose of antibiotics (Fenflor; AUV Veterinary Services B.V., Cuijk, the Netherlands) 1 mL/pig, intramuscular at day 1 and 3 of the experimental period and were placed in four disinfected rooms in a distinct part of the pig facility with a strict hygiene protocol (4). At the end of the experimental period, animals were subject to dissection for collection of colon digesta after EDTA blood samples were taken. The groups housed pigs (six per room) were fed *ad libitum* for 4–5 days prior to dissection for sample collection. At the dissection day, three pigs per room were euthanized on 1 day between 8.30 and 15.00 h to collect blood and digesta samples for further analysis. We used colon digesta and blood samples from pigs in the study that received a diet with a regular protein content [CP 168 g/kg; LSC (*n* = 18) and HSC (*n* = 18)].

**Table 1:** Ingredient and analyzed chemical composition of experimental diets fed to LSC and HSC pigs [data adapted from van der Meer et al. (12)].

**Diet components**	**Diet**
**Ingredient (g/kg of feed)**	
Wheat	308.6
Maize	200
Barley	200
Soybean meal	184.5
Maize starch	25.8
Sugarcane molasses	20
Limestone	14.7
Monocalcium phosphate	9
Soybean oil	19.2
Vitamin + mineral mix^a^	5
Salt	3.5
L-lysine HCL	3.4
Titanium dioxide	2.5
Sodium bicarbonate	2.1
L-threonine	1
L-tryptophan	0
DL-methionine	0.4
L-Valine	0.3
**Analyzed nutrients composition (g/kg)**	
NE (MJ/kg)^b^	9.8
DM	889.6
CP	166
Starch	474
Lys	9.8
Thr	6.6
Trp	2.2
Met + Cys	5.2
Ile	8.2
Arg	11.8
Phe	9.4
His	5.5
Leu	15
Tyr	6.2
Val	9.6

*LSC, low sanitary conditions; HSC, high sanitary conditions; NE, net energy; Lys, lysine; Thr, threonine; Trp, tryptophan; Met + Cys, methionine + cysteine; Ile, isoleucine; Arg, arginine; Phe, phenylalanine; His, histidine; Leu, leucine; Tyr, tyrosine; Val, valine*.

a *Supplied the following per kg of diet: 3.0 mg riboflavin, 20 mg niacine, 20 mg D-pantothenic acid, 10 mg choline chloride, 0.015 mg cyanocobalamin, 40 mg DL-α-tocopheryl acetate, 1.5 mg menadione, 6,000 IU retinyl acetate, 1,200 IU cholecalciferol, 0.2 mg folic acid, 1.0 mg thiamin, 1.0 mg pyridoxine HCl, 50 mg manganese oxide, 267 mg iron SO4·H2O, 60 mg copper SO4·5H2O, 140 mg zinc SO4·H2O, 0.44 mg disodium selenium trioxide, 1.0 mg potassium iodate*.

b *Based on chemical composition, digestibility and energy values for pigs from CVB (2011)*.

### Microbiome Composition Analysis

Colon digesta samples were handled as described previously for jejunum samples (22). Briefly, stored samples (−20°C) were thawed and mixed 1:1 with PBS, vortexed, and spun. After filtering steps, the QIAamp DNA Stool Mini Kit protocol was used as described by the manufacturer (https://www.qiagen.com/nl/resources/resourcedetail?id=df0aafde-ad92-4287-ad85-54cffb5fddc5&lang=en). Lastly, samples were eluted in the (provided) elute buffer and thereafter DNA quality was measured on the Nanodrop (Agilent Technologies).

Samples were sequenced by targeted-amplicon 16S sequencing, V3 and V4, on the MiSeq and analyzed for taxonomy profile per sample with clustering by profile by using QIIME (23). The QIIME pipeline has been described before (22). After sequencing we performed analyses of the summarized taxa at genus level and used the relative values enabling to compare the different samples. The diversity as described by the Shannon index (24) was calculated by the vegan package (25) within the R environment. Subsequently, we performed a Redundancy analysis (RDA) on the genus level of the microbiota data using the model: Y = Experimental treatment + error. Statistical significance testing for over- and underrepresentation of the bacterial groups was made at the genus level by performing the Wilcoxon signed-rank test, and *p*-values were converted to false discovery rate (FDR) values to correct for multiple testing.

### Metabolomics Sample Preparation and Analyses

Colon samples (*n* = 18 LSC, *n* = 18 HSC) were thawed and prepared for either the 1H Nuclear magnetic Resonance (NMR) or Triple quadrupole mass spectrometry (TQMS) analysis. In order to avoid pH differences in the sample (pH differences will cause shifts in NMR spectra) a sample of 1:1 diluted with NMR buffer (75 mM Na_2_HPO_4_ in 80%/20% H_2_O/D_2_O, pH 7.4; 500 μM maleic acid and 0.04% sodium azide) was used. For water soluble metabolites: 1 ml diluted NMR buffer was added to 0.5 gram colon material. Subsequently, the sample was vortexed and spun 60 min on the “Wheel,” and then centrifuged for 15 min at 21,100 g (room temperature). Additional purification was needed before analyzing these samples by NMR or TQMS using ultrafiltration with pre-washed with milliQ water Pall 3K Omega filters (OD003C34) (Pall corporation, Port Washington, NY, USA). Following centrifugation for 30 min at 10,000 g at room temperature 40 μl of the filtrate was used for TQMS and 200 μl for NMR. For the apolar metabolite fraction chloroform substituted water.

Of the same animals, EDTA blood serum samples were thawed, vortexed and centrifuged for 10 min at 21.100 g at 21°C. Additional purification was needed using ultrafiltration with pre-washed with milliQ water Pall 3K Omega filters (OD003C34). After the final washing step 200 μl NMR buffer was added on top of the filter and subsequently 200 μl serum was added and mixed. The samples were centrifuged at 10,000g (room temperature) for 45 min. For TQMS 40 μl was used, whereas for NMR 200 μl was used.

The 1H NMR spectra were acquired on a 600Bruker 600MHz Avance III 1H NMR spectrometer equipped with a CryoPlatform cryogenic cooling system, a BCU-05 cooling unit, and an ATM automatic tuning and matching unit (Bruker Biospin, Rheinstetten, Germany). The samples were measured in 3-mm 1H NMR tubes (Bruker matching system). Measurements were performed at a temperature of 27°C. Baseline corrections and zero alignment were performed manually for all spectra. The spectra were analyzed for background correction using machine-specific manufacturer software. The metabolites were identified using previous knowledge on the 1H NMR spectra.

The same samples were applied to TQMS analyzing over 50 pre-identified metabolites with high reliability, specificity, and accuracy and high quantitative measurements (26). The NMR and TQMS methods measure for the largest part different metabolites and the results are therefore complementary.

### Statistical Analyses and Bioinformatics

The NMR spectra were divided into bins of 0.04 ppm and the mean signal strength was used as an indicator. Differential signal strength related to the sanitary status was calculated with a Student's *t*-test. Biological information, such as annotation of the metabolites contributing to the discrimination of treatment groups, was retrieved from the human metabolome database [HMDB, http://www.hmdb.ca/; (27, 28)] and the KEGG database [https://www.genome.jp/kegg/; (29–31)], or manually determined based on the respective peaks. Data were presented as statistically significant differences between the sanitary groups. For all statistics: *p* < 0.05 was considered significant, with FDR < 0.05 and for the microbiota data an Average Relative Contribution (ARC) > 0.01%.

## Results

### Colon Microbiome Composition

The microbiota diversity in colon digesta was higher in LSC (2.99) compared to HSC (2.85) (Table 2: respectively, 30/35 and 4/35; *p* = 0.003). The abundance of 30 out of the 34 genera was higher in colon digesta from LSC pigs compared to HSC pigs (Table 2).

**Table 2:** Bacterial genera in colon differing in abundance between pigs under low (LSC) and high (HSC) sanitary conditions.

**Family**	**Genus**	***p*-value**	**FDR^**a**^**	**HSC^**b**^**	**LSC^**b**^**	**FC^**c**^ HSC/LSC**
Veillonellaceae	Megasphaera	3.0E-03	1.4E-02	1.93	1.15	1.68
Bifidobacteriaceae	Bifidobacterium	3.5E-06	3.8E-05	0.15	0.11	1.42
Rikenellaceae		2.5E-05	1.9E-04	0.02	0.02	1.26
Ruminococcaceae	Oscillospira	6.4E-03	2.7E-02	2.70	2.67	1.01
WCHB1.25		8.1E-04	4.9E-03	0.01	0.01	0.97
Lachnospiraceae		7.4E-06	7.2E-05	5.07	5.54	0.92
Porphyromonadaceae	Paludibacter	1.8E-07	3.8E-06	0.03	0.04	0.79
Helicobacteraceae	Helicobacter	9.9E-07	1.5E-05	0.01	0.01	0.77
Helicobacteraceae	Flexispira	1.9E-06	2.3E-05	0.02	0.02	0.77
Lachnospiraceae	Lachnospira	2.6E-03	1.3E-02	0.36	0.47	0.75
Erysipelotrichaceae	p.75.a5	6.0E-08	3.8E-06	0.08	0.11	0.73
Tissierellaceae		1.2E-02	4.4E-02	0.36	0.50	0.71
Alphaproteobacteria^d^		3.7E-03	1.7E-02	0.06	0.08	0.71
Veillonellaceae		1.7E-07	3.8E-06	0.06	0.09	0.68
Veillonellaceae	Anaerovibrio	4.7E-08	3.8E-06	0.08	0.12	0.63
Lachnospiraceae	Shuttleworthia	1.4E-07	3.8E-06	0.72	1.24	0.58
Sphaerochaetaceae	Sphaerochaeta	8.7E-04	5.1E-03	0.12	0.20	0.58
Lachnospiraceae	Butyrivibrio	1.7E-07	3.8E-06	0.06	0.11	0.56
Anaeroplasmataceae	Anaeroplasma	5.7E-03	2.5E-02	0.01	0.01	0.54
BS11		7.9E-03	3.2E-02	0.01	0.01	0.54
Veillonellaceae	Acidaminococcus	6.6E-08	3.8E-06	0.08	0.18	0.45
Veillonellaceae	Dialister	8.8E-08	3.8E-06	0.22	0.51	0.43
Deltaproteobacteria^d^		2.7E-07	5.3E-06	0.05	0.11	0.39
Elusimicrobiaceae		6.2E-06	6.3E-05	0.01	0.02	0.37
Fibrobacteraceae	Fibrobacter	1.8E-07	3.8E-06	0.01	0.04	0.37
RF3^d^		9.5E-03	3.8E-02	0.01	0.02	0.28
WPS.2^e^		2.0E-06	2.3E-05	0.02	0.07	0.25
RF16		6.6E-07	1.1E-05	0.02	0.08	0.19
Succinivibrionaceae	Succinivibrio	1.6E-03	8.7E-03	0.14	0.78	0.18
Mogibacteriaceae	Mogibacterium	1.8E-05	1.5E-04	0.01	0.08	0.18
Deferribacteraceae	Mucispirillum	3.5E-07	6.1E-06	0.03	0.18	0.17
Betaproteobacteria^d^		5.2E-04	3.5E-03	0.00	0.09	0.04
TA18^d^		1.9E-03	9.8E-03	0.00	0.01	0.03
Brachyspiraceae	Brachyspira	8.1E-04	4.9E-03	0.00	0.04	0.02

a *False discovery rate (FDR)*.

b *Low sanitary condition (LSC) and high sanitary condition (HSC) in relative intensities (i.e., signal intensities)*.

c *Fold change (FC)*.

d *This bacterial group could only be classified on Class level*.

e *This bacterial group could only be classified on Phylum level*.

*The data are ordered for decreasing fold change*.

**Figure 1:**
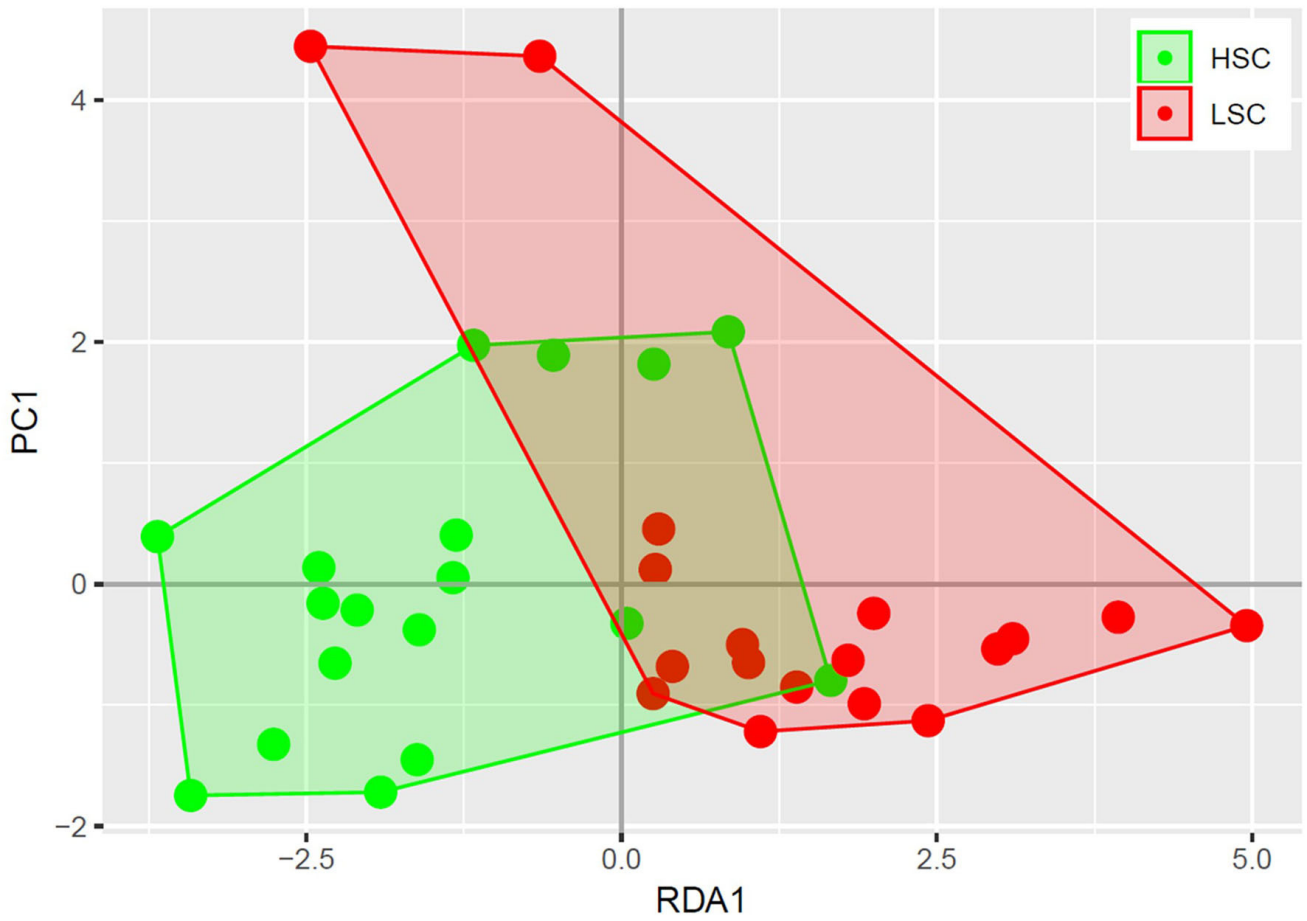
Redundancy analysis of colon bacterial composition. Each circle represents a colon sample of a piglet, where high sanitary conditions (HSC) are depicted in red and low sanitary conditions (LSC) in green. The bacterial composition of HSC and LSC differ (*p* < 0.05).

A higher abundance was observed for four bacterial genera in digesta of HSC compared to LSC pigs. One of them being a member of the *Megasphaera*, a genus involved in lactate fermentation. For more detailed information one is referred to the Supplementary Information 1. A redundancy analysis (RDA, Figure 1) shows that the microbiota composition in colon at genus level differs between LSC and HSC pigs (*p* < 0.05). The first component (RDA1) comprises the largest difference between the two experimental groups.

### Colon Metabolome Composition Related to Sanitary Conditions

Table 3 shows a list of NMR identified metabolites in colon digesta with different abundance between pigs kept under LSC or HSC conditions. Metabolite intensities were corrected for sample weights. All metabolites were derived from the polar metabolite analysis. In total 25, of which 8 putative, metabolites were different between both groups. All metabolites were higher in HSC compared to LSC pigs, probably due to more uniform metabolite patterns observed in HSC animals. The higher variability among LSC pigs resulted in a more disperse pattern with more “chemical noise.” The apolar metabolite analysis showed three NMR bins with no reliable information on their identity available (unknown peaks only–data not shown). Supplementary Information 2 provides available biological information for the discriminating metabolites and provides biological processes in which these metabolites are involved. The metabolites can be related to a wide range of biological processes including general cellular (energy) metabolism, RNA/DNA metabolism and functioning, protein and lipid modification, muscle metabolism (e.g., leucine and creatinine) and body protein deposition and [e.g., adenosine and nicotinic acid (niacin)], renal health and muscle energy metabolism (creatinine), immune stimulation (e.g., dimethylglycine and adenosine), (synthesis of) neurotransmitters (glutamine, valeric acid, and cytidine), mitochondrial disease (dimethylglycine and 5-hydroxymethyl uracil), and energy supply of colon cells. Table 4 shows the results of the TQMS analysis of colon digesta.

**Table 3:** NMR metabolites with differential colonic levels between pigs under high (HSC) and low (LSC) sanitary conditions.

**Metabolite**	**ppm^**a**^**	***p*-value**	**HSC^**b**^**	**LSC^**c**^**	**FC HSC/LSC**
Inosine	8.22	0.019	1,569,004	1,262,322	1.24
Urocanic acid (overlaps with uridine)	7.86	0.024	1,507,445	1,219,191	1.24
Cytidine	7.82	0.030	1,272,221	1,090,030	1.17
Uracil-derivative	7.66	0.001	1,051,337	755,404	1.39
Phenylalanine	7.38	0.001	5,326,128	3,849,696	1.38
Phenylalanine	7.30	0.006	8,785,567	6,387,726	1.38
Gallic acid	7.02	0.003	1,758,394	1,192,498	1.47
Gentisic acid	6.98	0.000	3,135,842	1,958,977	1.60
Cresol	6.94	0.001	2,622,976	1,760,954	1.49
Tyrosine	6.90	0.002	2,908,448	1,849,886	1.57
Fumaric acid	6.54	0.042	678,028	525,169	1.29
Orotic acid	6.18	0.004	423,688	294,373	1.44
Inosine derivative	6.10	0.006	571,384	418,067	1.37
Maleic acid	6.02	0.000	1,369,948	1,154,823	1.19
Uracil	5.70	0.001	526,920	378,035	1.39
Glucosamine (tentative)	5.54	0.049	1,014,960	841,786	1.21
Sucrose	5.42	0.034	734,954	597,302	1.23
Asparagine	2.90	0.000	8,647,401	6,296,110	1.37
Asparagine	2.86	0.008	10,288,172	8,449,148	1.22
Succinate	2.42	0.000	16,927,583	11,079,148	1.53
Glutamic acid	2.38	0.015	33,766,106	24,645,419	1.37
Hydroxybutyric acid	2.34	0.011	36,311,422	29,279,850	1.24
n-valerate	1.58	0.005	17,909,947	13747776	1.30
Butyrate	1.54	0.004	81,290,463	58,211,276	1.40
Butyrate	0.90	0.000	68,198,492	46,297,287	1.47

a *Bin number of the NMR spectra*.

b *High sanitary condition*.

c *Low sanitary condition*.

*Metabolite intensities were corrected for sample weight. All metabolites are higher in HSC then in LSC pigs. Metabolites are ranked by concentration*.

**Table 4:** TQMS metabolites with differential colonic levels between pigs under high (HSC) and low (LSC) sanitary conditions.

**Metabolite**	***p*-value**	**HSC^**a**^**	**LSC^**b**^**	**FC^**c**^ HSC/LSC**
Nicotinic acid (niacin)	0.009	1,151,213	680,230	1.69
Histamine	0.009	7,185,303	2,507,969	2.86
Histidine	0.021	846,159	1,367,104	0.62

a *High sanitary condition*.

b *Low sanitary condition*.

c *Fold change*.

*The data were ranked by P-value of the HSC-LSC difference*.

### Blood Metabolome Composition Related to Sanitary Status

Table 5 presents the NMR blood plasma metabolites showing differences between LSC and HSC managed pigs. A number of significant peak intervals represents unknown metabolites. The annotated metabolites represent several AAs and metabolites related to energy metabolism, DNA/RNA metabolism, methylation/detoxification (e.g., betaine), and general metabolism. The results showed that all metabolites were higher in HSC than in LSC pigs with the exception of the two alcohols, ethanol and propanediol. For detailed biological information about the metabolites see Supplementary Information 2. Table 6 shows the results of the TQMS analysis of blood samples. The two methods measure different metabolites and the results are therefore complementary, adding three specific metabolites to the discriminating compounds being pantothenic acid, methionine, and niacinamide.

**Table 5:** NMR Metabolites with differential blood serum levels between pigs under high (HSC) and low (LSC) sanitary conditions.

**Metabolite**	**^**a**^ppm**	***p*-value**	**HSC^**b**^**	**LSC^**c**^**	**FC HSC/LSC**
Phenylalanine	7.18	0.020	1,075,542	899,435	1.20
Tyrosine	6.90	0.030	1,092,761	939,066	1.16
Betaine	3.26	0.022	29,495,896	26,123,568	1.13
Ornithine	3.06	0.016	1,327,174	1,073,002	1.24
Creatine	3.02	0.002	6,759,282	5,450,382	1.24
Methionine	2.62	0.005	408,694	283,248	1.44
Valine	2.26	0.013	1,535,304	1,355,263	1.13
Acetate	1.90	0.000	7,192,506	5,801,785	1.24
Lysine	1.74	0.004	3,201,914	2,605,699	1.23
Lysine/arginine	1.70	0.001	3,596,604	2,856,496	1.26
Arginine	1.66	0.003	1,564,087	1,338,525	1.17
Ethanol	1.18	0.017	3,574,504	5,078,033	0.70
Propanediol	1.14	0.023	9,856,413	13,048,687	0.76
Valine	1.02	0.014	5,039,859	4,407,670	1.14
Leucine	0.94	0.005	7,782,478	6,669,890	1.17

a *Integral of the NMR spectra*.

b *High sanitary condition*.

c *Low sanitary condition*.

*Metabolites are ranked by concentration*.

**Table 6:** TQMS Metabolites with differential blood serum levels between pigs managed under high (HSC) and low (LSC) sanitary conditions.

**Metabolite**	***p*-value**	**HSC^**a**^**	**LSC^**b**^**	**FC^**c**^ HSC/LSC**
Pantothenic acid	0.019	235,288	161,114	1.46
Methionine	0.048	1,932,497	1,477,238	1.31
Niacinamide (nicotinamide)	0.048	32,886	24,278	1.35

a *High sanitary condition*.

b *Low sanitary condition*.

c *Fold change*.

*The data were ranked by P-value of the HSC-LSC difference*.

## Discussion

Livestock management conditions greatly affect pig performance. Among management factors such as environmental temperature, availability of space, diet composition and access to feed, sanitary conditions are of importance. We created a sanitary status model with the aim to create differences in degree of immune system activation and growth performance between pigs and measure aspects of whole body energy metabolism (4, 12). All pigs were clinically healthy. The LSC pigs showed a greater haptoglobin and lower CRP concentration in blood serum than HSC pigs (13). LSC may activate the immune system and induce stress, thereby affecting the (subclinical) health of the animal, possibly affecting nutrient requirements and appetite of the pigs (7, 32). This study describes the effects of a model intervention to change the sanitary status in the housing of pigs, including the regular spread of fresh manure of pigs from different farms. Although the detailed chemical and microbial composition of the manure, including the potential presence of pathogens, was not characterized in the present study, a clear overall effect of the sanitary status challenge on the growth performance of the pigs was observed. The BW at the day of sacrifice was lower for LSC (28.4 ± 0.8 kg) compared with HSC pigs (38.0 ± 0.8 kg, *P* ≤ 0.05) (4, 12). Substantial differences in growth performance of pigs can also be noticed between farms in practice with often no clear and detailed explanation for the background of these differences. The differences in body weight between experimental groups of pigs were induced by the sanitary status protocol applied to both groups. In the present manuscript we compare metabolome profiles of blood and digesta of both groups. As a result the direct effect of differences in mean body weight on the response parameters (metabolome profiles) cannot be evaluated. The range in body weight of pigs within treatments groups was relatively small and do not allow for evaluation of the effects of body weight on the metabolome profiles within treatment. In addition, LSC pigs had a higher fasting heat production as a proxy for energy requirements for maintenance than HSC pigs (12). We investigated the composition of the colon microbiome and differences in metabolite composition in the colon and blood plasma as affected by exposure to different sanitary conditions and related differences in nutrient metabolism in the same animals. Our results indicate that sanitary conditions affect both the colonic microbiome composition and diversity, and the composition of the metabolome in colon and blood.

We used NMR and TQMS as methods for analyzing the metabolome. These methods differ because the NMR is a wide screening method while the TQMS is dedicated to measure predefined metabolites with high annotation certainty. Furthermore, the error of measurement using NMR is smaller than of TQMS. As a consequence NMR is able to detect smaller differences in concentrations of metabolites than TQMS. These methodological differences explain the differences between methodologies in the number of metabolites identified to discriminate the metabolomes of LSC and HSC pigs. Finally, it should be noted that, with the exception of the KEGG database, the databases include mainly contain human-derived data. Although in general pigs and humans have a highly similar physiology (33), the former may impact the results.

### Colon Microbiome Composition

High sanitary conditions reflecting the health status of animals is often accompanied by a high gut microbiome diversity (34, 35), especially in adult animals (36). Indeed we observed a different colon microbiota composition between pigs kept in contrasting sanitary conditions. Redundancy analysis showed that the colon microbiota composition of the pigs managed under the two sanitary statuses greatly differed. This may suggest that the sanitary status regulate biological mechanisms differently resulting in differences in metabolome composition. Furthermore, as gut microbiota composition has been related to productivity traits such as growth rate (37), this may indicate a biological or functional relationship between gut health, immune and productivity traits. In our experiments LSC and HSC pigs differed for nutrient digestibility, with the HSC animals showing slightly higher digestibility. This may relate to the differential colon microbiota composition. The range in body weight of pigs with treatments groups was relatively small and did not allow for evaluation of the effects of body weight on the metabolome profiles within treatment.

To investigate which bacterial groups differed between the sanitary conditions groups we observed four bacterial genera having a higher relative abundance in HSC vs. LSC pigs, including the *Megasphaera* genus. The *Megasphaera* are a genus of *Firmicutes* bacteria classified within the class *Negativicutes*, probably a member of the *Clostridia* (38). Other *Megasphaera* species, however, are obligate anaerobic bacteria typically isolated from feces (39). The *Megasphaera* are involved in lactate fermentation. Jiang et al. (40) showed that *Megasphaera* may have a potential advantage in the alleviation of D-lactic acidosis in the gut of pigs (41). Clinically, D-lactic acidosis is characterized by episodes of metabolic acidosis, which is thought to be due to the absorption of D-lactic acid and other unidentified chemicals produced by bacterial fermentation in the colon. This suggests that this bacterial genus is related to certain aspects of gut health and may explain why these bacteria showed a higher abundance under HSC conditions. Van der Meer et al. (12) showed that the dietary requirements for the AAs methionine, threonine, and tryptophan, relative to lysine, depend on the sanitary conditions. Furthermore, sanitary conditions were also related to differences in the concentration of white blood cells indicating gut health and general health effects.

The strict anaerobic bacteria *Lachnospiraceae*, a family of the order *Clostridiales*, constitutes one of the major taxonomic groups of the gut microbiota involved in SCFA synthesis from fermentation of complex polysaccharides. The *Lachnospiraceae* (4 out of 34 genera) are a family of the order *Clostridiales*, strict anaerobic bacteria capable of degrading complex polysaccharides to short-chain fatty acids (SCFAs) (42). The SCFA include acetate, butyrate, and propionate. Furthermore, the *Lachnospiraceae* favor hindgut fermentation, which contributes to the energy supply of the host. In general, herbivores having a higher abundance of this family in the digestive tract than carnivores (43), while pigs are considered omnivores, while receiving diets largely based on plant derived ingredients. The wide range of functions carried out by *Lachnospiraceae* may influence their relative abundance in gut communities. These bacteria are among the most abundant taxa in the microbiota (44, 45). The *Lachnospiraceae* ferment diverse plant polysaccharides to short-chain fatty acids (butyrate, acetate) and ethanol (46). In human adults, members of this family have been associated with protection against *C. difficile* infections and obesity (43).

### Colon and Blood Metabolome

Differential abundance of colon metabolites indicated in most cases a higher abundance in HSC compared to LSC managed pigs. In most cases the differences were relatively small. As NMR is able to measure ratio differences as small as 1 to 1.4, however, these differences can be considered as reliable. While the biological meaning of such differences in colon digesta may be uncertain, such differences in the blood, being subject to homeostatic regulation, must be considered biologically important. Two TQMS detected metabolite differences were larger between groups than others: niacin and histamine. Niacin, also known as vitamin B3, is a precursor for NAD+. Dietary niacin may be taken up from the diet or in part synthesized by the microbiome in the gut (47). Niacin shortage in humans leads to the pellagra disease marked by dementia, diarrhea, and dermatitis. If left untreated, pellagra can be fatal (48). Niacin deficiency in commercial kept pigs is unlikely as niacin and other vitamins are supplemented to the diets in sufficient quantities. Histamine, likely resulting from protein fermentation or bacterial secretion in the gut is involved in inflammatory responses and regulates physiological functions in the gut by acting as a messenger that interacts with several cellular targets (49–52). The higher level in HSC pigs as compared to LSC pigs was unexpected. The AA histidine is also a precursor of histamine showed higher levels in LSC pigs compared to HSC pigs in both colon digesta and blood. This could probably be linked to the hygiene hypothesis: cleaner environments in early life lead to more asthmatic symptoms, like overreaction by histamine. However, this differences was unexpected and needs further evaluation and explanation.

The HSC managed pigs showed higher abundance of five free AAs in colon digesta probably derived from fermentation of dietary and/or endogenous proteins, which may also give rise to the formation of N-containing end products such as ammonia. Van der Meer et al. (12) showed that HSC pigs showed a higher fecal protein and energy digestibility compared to LSC pigs, suggesting that enzymatic protein digestibility may be reduced in LSC pigs and protein fermentation enhanced.

Blood metabolites related to energy metabolism and DNA/RNA metabolism may be synthesized by the gut microbiome and absorbed by the intestinal tissue and transferred to the blood circulation. Contrasts for these metabolites may relate to variation in animal growth in general (53), probably via regulation of appetite (54), and muscle growth and protein accretion in particular. We can speculate that this may relate to the underlying biological mechanisms for the observed growth differences between both groups of animals (12). Further biological analysis, such as functional annotation of the metabolites, indicates that metabolites related to immune traits are contributing to the discrimination of both sanitary condition groups, with specific metabolites being lower in LSC pigs. This may suggest that the LSC pigs have a lower ability to regulate a “healthy gut” microbiome composition (i.e., a diverse composition regulating a variety of biological functions). Two short chain fatty acids (SCFAs), one of which is the preferred energy source of colon cells (55) (butyrate) were slightly higher in abundance in LSC pigs. The biological meaning of this observation is not clear, also considering the small size of the difference. However, as mentioned before, NMR enables to measure small differences quantitatively.

Next to their important roles in protein synthesis L-glutamine and valeric acid have been indicated as precursors of the neurotransmitters glutamate and GABA, respectively. These neurotransmitters are remarkably different in function as glutamate is a widely expressed stimulatory neurotransmitter while GABA is a widely expressed inhibitory neurotransmitter downregulating neuron excitability (56, 57). Interestingly the first showed higher abundance in the HSC managed pigs while the latter showed the opposite pattern. In general both neurotransmitters may therefore stimulate neural activity differently. However, it should be noted that these metabolites were found in the colon digesta. Such metabolites are likely only relevant for the animal if absorbed into the systemic blood in physiologically relevant quantities.

The blood metabolome partly refers to the colon metabolome since it also contains metabolites absorbed from the gut. However, metabolites may also be related to cell metabolism in organs and tissues. As for colon metabolites, most blood metabolites were found with higher abundance in pigs managed under HSC than in pigs managed under LSC. The higher niacin concentration in blood of HSC pigs confirms results observed in colon digesta. This suggests that the HSC pigs have potentially more NAD^+^ and may have a higher metabolic activity: multi-organ decrease of NAD^+^ has been related to aging and related diseases (58), DNA repair and mitochondrial maintenance (59). Therefore, our results suggest that sanitary conditions may affect animal health and whole body metabolism.

The annotated discriminating metabolites in blood include five AAs, two of them shared with results observed for the colonic digesta composition. This may suggest a direct relationship between blood and gut metabolite levels. These results suggest that the sanitary conditions influence the animals' metabolism, specifically related to protein synthesis and degradation. The metabolites related to energy metabolism and DNA/RNA metabolism found to be affected by sanitary condition were all different for blood and colon digesta suggesting that differences in blood metabolites were mainly related to differences in host metabolism.

Interestingly, two alcohols, ethanol and propanediol, showed higher levels in LSC pigs as compared with HSC pigs. Microbial fermentation of glycerol synthesizes propanediol, with *Clostridia* and *Enterobacteriaceae* as important sources (60). We did not observe differences for these bacteria in colon digesta between LSC and HSC pigs. Therefore, differential nutrient uptake from the gut by LSC and HSC pigs may explain the differences between colon and blood serum metabolite content. In humans alcohols are not considered healthy and this may also point to the influence of the sanitary conditions on these pigs.

### Can the Response of Pigs to (Sanitary) Stress Be Detected in Feces?

Environmental stresses greatly influences livestock production and health. But how to determine the effects of such stressors in pigs using non-invasive and non-stress causing methods, and how to select animals which are coping best with such stress? We specifically investigated sanitary stress because there is a large range of sanitary conditions on commercial farms. Providing farmers with the best suitable animal type will increase animal health and welfare, and farm productivity. We showed that metabolite abundances in colon digesta and blood of pigs differ when keeping them in two rather extreme experimental sanitary conditions. Some of the differences could be related biologically to other results (growth performance, nutrient metabolism, nutrient digestion and subclinical health status) obtained in the study. Although the metabolomes of the colon digesta and blood clearly differ, some overlap was also noted as well. Our results point to biological mechanisms which may underlie pig growth rate differences under these sanitary conditions. As such, the biological mechanisms relate the metabolites to the sanitary conditions. More research is needed to confirm these effects under less extreme commercial conditions before we can conclude on the usefulness of these metabolites as biomarkers for productivity, health, and welfare in relation to sanitary conditions.

## Data Availability Statement

All datasets generated for this study are included in the article/Supplementary Material.

## Ethics Statement

The animal study was reviewed and approved by Animal Care and Use Committee of Wageningen University, the Netherlands.

## Author Contributions

MP: analyzed the metabolomics data and wrote the manuscript in consultation with AJ, YM, LK, JV, and DS. AJ: received funding for the animal experiment, coordinated the animal experiment, and made the samples available. LK: performed the metabolomics analysis. YM: performed the animal experiment and analyzed the data, made the samples available. JV: performed the metabolomics analysis and analyzed the raw data. DS: received funding for the metabolomics analysis, coordinated and performed (part of the) metabolomics data analysis. All authors: contributed to the article and approved the submitted version.

## Conflict of Interest

The authors declare that the research was conducted in the absence of any commercial or financial relationships that could be construed as a potential conflict of interest.

## Supplementary Material

Supplementary Information 1 Details of the colon digesta microbiota composition.

Supplementary Information 2 Biological information about the metabolites with different levels between pigs managed under high sanitary conditions or low sanitary conditions.
