# Supplementary material for: Sanitary Conditions Affect the Colonic Microbiome and the Colonic and Systemic Metabolome of Female Pigs
Source: Front Vet Sci. 2020 Oct 26;7:585730. doi: 10.3389/fvets.2020.585730 (PMC7649119; doi:10.3389/fvets.2020.585730)
Supplement: Supplementary file 1 [file Table_1.DOCX]

| **Bacterial Group** | **p.value** | **FDR** | **HSC** | **LSC** | **FC HSC/LSC** |
| --- | --- | --- | --- | --- | --- |
| k__Bacteria.p__Firmicutes.c__Clostridia.f__Veillonellaceae.g__Megasphaera | 0.002988648 | 0.013733551 | 1.930 | 1.151 | 1.676223376 |
| k__Bacteria.p__Actinobacteria.c__Actinobacteria.f__Bifidobacteriaceae.g__Bifidobacterium | 3.52E-06 | 3.77E-05 | 0.149 | 0.105 | 1.416966498 |
| k__Bacteria.p__Bacteroidetes.c__Bacteroidia.f__Rikenellaceae.g__ | 2.50E-05 | 0.000192863 | 0.019 | 0.015 | 1.256635901 |
| k__Bacteria.p__Firmicutes.c__Clostridia.f__Ruminococcaceae.g__Oscillospira | 0.006402233 | 0.026861542 | 2.701 | 2.671 | 1.011395318 |
| *k__Bacteria.p__Verrucomicrobia.c__Verruco.5.f__WCHB1.25.g__* | *0.00081122* | *0.00489267* | *0.010* | *0.010* | *0.97076294* |
| k__Bacteria.p__Firmicutes.c__Clostridia.f__Lachnospiraceae.g__ | 7.42E-06 | 7.17E-05 | 5.071 | 5.541 | 0.915216354 |
| k__Bacteria.p__Bacteroidetes.c__Bacteroidia.f__Porphyromonadaceae.g__Paludibacter | 1.76E-07 | 3.77E-06 | 0.034 | 0.043 | 0.789362815 |
| *k__Bacteria.p__Proteobacteria.c__Epsilonproteobacteria.f__Helicobacteraceae.g__Helicobacter* | *9.86E-07* | *1.46E-05* | *0.008* | *0.011* | *0.77461775* |
| k__Bacteria.p__Proteobacteria.c__Epsilonproteobacteria.f__Helicobacteraceae.g__Flexispira | 1.92E-06 | 2.28E-05 | 0.019 | 0.024 | 0.773475232 |
| k__Bacteria.p__Firmicutes.c__Clostridia.f__Lachnospiraceae.g__Lachnospira | 0.002641983 | 0.013074428 | 0.355 | 0.471 | 0.754205921 |
| k__Bacteria.p__Firmicutes.c__Erysipelotrichi.f__Erysipelotrichaceae.g__p.75.a5 | 5.99E-08 | 3.77E-06 | 0.084 | 0.114 | 0.734994192 |
| k__Bacteria.p__Firmicutes.c__Clostridia.f__.Tissierellaceae..g__ | 0.011839117 | 0.043941338 | 0.356 | 0.498 | 0.71399341 |
| k__Bacteria.p__Proteobacteria.c__Alphaproteobacteria.f__.g__ | 0.003714115 | 0.016670328 | 0.058 | 0.081 | 0.709450908 |
| k__Bacteria.p__Firmicutes.c__Clostridia.f__Veillonellaceae.g__ | 1.66E-07 | 3.77E-06 | 0.062 | 0.091 | 0.684035056 |
| k__Bacteria.p__Firmicutes.c__Clostridia.f__Veillonellaceae.g__Anaerovibrio | 4.72E-08 | 3.77E-06 | 0.077 | 0.121 | 0.634536375 |
| k__Bacteria.p__Firmicutes.c__Clostridia.f__Lachnospiraceae.g__Shuttleworthia | 1.39E-07 | 3.77E-06 | 0.722 | 1.242 | 0.581634468 |
| k__Bacteria.p__Spirochaetes.c__Spirochaetes.f__Sphaerochaetaceae.g__Sphaerochaeta | 0.000865277 | 0.005060557 | 0.116 | 0.199 | 0.580703489 |
| k__Bacteria.p__Firmicutes.c__Clostridia.f__Lachnospiraceae.g__Butyrivibrio | 1.66E-07 | 3.77E-06 | 0.059 | 0.106 | 0.557849675 |
| *k__Bacteria.p__Tenericutes.c__Mollicutes.f__Anaeroplasmataceae.g__Anaeroplasma* | *0.00573051* | *0.0245775* | *0.006* | *0.011* | *0.54433234* |
| *k__Bacteria.p__Bacteroidetes.c__Bacteroidia.f__BS11.g__* | *0.00788659* | *0.03238536* | *0.007* | *0.014* | *0.53691945* |
| k__Bacteria.p__Firmicutes.c__Clostridia.f__Veillonellaceae.g__Acidaminococcus | 6.62E-08 | 3.77E-06 | 0.081 | 0.180 | 0.449228613 |
| k__Bacteria.p__Firmicutes.c__Clostridia.f__Veillonellaceae.g__Dialister | 8.84E-08 | 3.77E-06 | 0.219 | 0.508 | 0.430524008 |
| k__Bacteria.p__Proteobacteria.c__Deltaproteobacteria.f__.g__ | 2.74E-07 | 5.30E-06 | 0.045 | 0.114 | 0.393548555 |
| *k__Bacteria.p__Elusimicrobia.c__Elusimicrobia.f__Elusimicrobiaceae.g__* | *6.22E-06* | *6.31E-05* | *0.008* | *0.022* | *0.36936803* |
| k__Bacteria.p__Fibrobacteres.c__Fibrobacteria.f__Fibrobacteraceae.g__Fibrobacter | 1.76E-07 | 3.77E-06 | 0.013 | 0.035 | 0.367134461 |
| *k__Bacteria.p__Tenericutes.c__RF3.f__.g__* | *0.00952073* | *0.03825128* | *0.006* | *0.020* | *0.27717107* |
| k__Bacteria.p__WPS.2.c__.f__.g__ | 2.01E-06 | 2.28E-05 | 0.016 | 0.065 | 0.251136945 |
| k__Bacteria.p__Bacteroidetes.c__Bacteroidia.f__RF16.g__ | 6.60E-07 | 1.06E-05 | 0.015 | 0.079 | 0.187706713 |
| k__Bacteria.p__Proteobacteria.c__Gammaproteobacteria.f__Succinivibrionaceae.g__Succinivibrio | 0.001569693 | 0.008655735 | 0.143 | 0.775 | 0.184929 |
| k__Bacteria.p__Firmicutes.c__Clostridia.f__.Mogibacteriaceae..g__Mogibacterium | 1.75E-05 | 0.000146172 | 0.014 | 0.080 | 0.178785383 |
| k__Bacteria.p__Deferribacteres.c__Deferribacteres.f__Deferribacteraceae.g__Mucispirillum | 3.46E-07 | 6.06E-06 | 0.031 | 0.180 | 0.169749342 |
| *k__Bacteria.p__Proteobacteria.c__Betaproteobacteria.f__.g__* | *0.00052213* | *0.00347484* | *0.004* | *0.091* | *0.03875499* |
| *k__Bacteria.p__Proteobacteria.c__TA18.f__.g__* | *0.00188289* | *0.00982157* | *0.000* | *0.012* | *0.03329* |
| *k__Bacteria.p__Spirochaetes.c__.Brachyspirae..f__Brachyspiraceae.g__Brachyspira* | *0.00081122* | *0.00489267* | *0.001* | *0.036* | *0.01633072* |
