# Supplementary material for: Sanitary Conditions Affect the Colonic Microbiome and the Colonic and Systemic Metabolome of Female Pigs
Source: Front Vet Sci. 2020 Oct 26;7:585730. doi: 10.3389/fvets.2020.585730 (PMC7649119; doi:10.3389/fvets.2020.585730)
Supplement: Supplementary Information 1 — Details of the colon digesta microbiota composition. [file Data_Sheet_1.docx]

Supplementary data

Differentially expressed blood en Colon metabolites.

**Blood metabolites**

| **Interval** | **P-value** | **High_avg** | **Low_avg** | **FC H/L** | **Metabolite** |
| --- | --- | --- | --- | --- | --- |
| 7.18 | 0.020 | 1075542 | 899435 | 1.20 | phenylalanine |
| 6.90 | 0.030 | 1092761 | 939066 | 1.16 | tyrosine |
| 3.26 | 0.022 | 29495896 | 26123568 | 1.13 | tyrosine |
| 3.06 | 0.016 | 1327174 | 1073002 | 1.24 | betaine |
| 3.02 | 0.002 | 6759282 | 5450382 | 1.24 | creatine |
| 2.62 | 0.005 | 408694 | 283248 | 1.44 | unknown |
| 2.26 | 0.013 | 1535304 | 1355263 | 1.13 | valine |
| 1.94 | 0.005 | 2079683 | 1768147 | 1.18 | noise |
| 1.90 | 0.000 | 7192506 | 5801785 | 1.24 | acetate |
| 1.74 | 0.004 | 3201914 | 2605699 | 1.23 | thymine? |
| 1.70 | 0.001 | 3596604 | 2856496 | 1.26 | unknown |
| 1.66 | 0.003 | 1564087 | 1338525 | 1.17 | unknown |
| 1.18 | 0.017 | 3574504 | 5078033 | 0.70 | unknown |
| 1.14 | 0.023 | 9856413 | 13048687 | 0.76 | unknown |
| 1.02 | 0.014 | 5039859 | 4407670 | 1.14 | valine |
| 0.94 | 0.005 | 7782478 | 6669890 | 1.17 | leucine |
| 0.74 | 0.023 | 11674 | 87360 | 0.13 | unknown |

**Colon metabolites**

| **Colon** | **Match** | **HMDB** | **PubChem** | **KEGG** | **FC HSC/LSC** |
| --- | --- | --- | --- | --- | --- |
| acetate | Acetic acid | HMDB0000042 | 176 | C00033 | 0.75 |
| adenosine | Adenosine | HMDB0000050 | 60961 | C00212 | 0.9 |
| butyrate | Butyric acid | HMDB0000039 | 264 | C00246 | 0.97 |
| Creatinine | Creatinine | HMDB0000562 | 588 | C00791 | 1.37 |
| Dimethylglycine | Dimethylglycine | HMDB0000092 | 673 | C01026 | 1.22 |
| Fumarate | Fumaric acid | HMDB0000134 | 444972 | C00122 | 1.24 |
| Glucosamine | Glucosamine | HMDB0001514 | 439213 | C00329 | 1.15 |
| Glutamine | L-Glutamine | HMDB0000641 | 5961 | C00064 | 1.53 |
| inosine | Inosine | HMDB0000195 | 6021 | C00294 | 1.24 |
| isoleucine | L-Isoleucine | HMDB0000172 | 6306 | C00407 | 0.55 |
| Leucine | L-Leucine | HMDB0000687 | 6106 | C00123 | 0.55 |
| Lysine | L-Lysine | HMDB0000182 | 5962 | C00047 | 1.12 |
| maleic acid | Maleic acid | HMDB0000176 | 444266 | C01384 | 1.19 |
| methionine | L-Methionine | HMDB0000696 | 6137 | C00073 | 1.12 |
| nicotinic acid | Nicotinic acid | HMDB0001488 | 938 | C00253 | 1.18 |
| Phenylalanine | L-Phenylalanine | HMDB0000159 | 6140 | C00079 | 1.35 |
| propionate | Propionic acid | HMDB0000237 | 1032 | C00163 | 0.97 |
| Tyrosine | L-Tyrosine | HMDB0000158 | 6057 | C00082 | 1.33 |
| valeric acid | Valeric acid | HMDB0000892 | 7991 | C00803 | 0.97 |
| valine | L-Valine | HMDB0000883 | 6287 | C00183 | 0.55 |
| Xanthine | Xanthine | HMDB0000292 | 1188 | C00385 | 1.05 |
